# *Trypanosoma cruzi* IIc: Phylogenetic and Phylogeographic Insights from Sequence and Microsatellite Analysis and Potential Impact on Emergent Chagas Disease

**DOI:** 10.1371/journal.pntd.0000510

**Published:** 2009-09-01

**Authors:** Martin S. Llewellyn, Michael D. Lewis, Nidia Acosta, Matthew Yeo, Hernan J. Carrasco, Maikell Segovia, Jorge Vargas, Faustino Torrico, Michael A. Miles, Michael W. Gaunt

**Affiliations:** 1 London School of Hygiene and Tropical Medicine, London, United Kingdom; 2 Departamento de Medicina Tropical, Instituto de Investigaciones en Ciencias de la Salud, Universidad Nacional de Asuncion, Paraguay; 3 Instituto de Medicina Tropical, Universidad Central de Venezuela, Los Chaguaramos, Caracas, Venezuela; 4 Centro Nacional de Enfermedades Tropicales, Santa Cruz, Bolivia; 5 Universidad Mayor de San Simon, Cochabamba, Bolivia; Universidad Centroamericana, Nicaragua

## Abstract

*Trypanosoma cruzi*, the etiological agent of Chagas disease, is highly genetically diverse. Numerous lines of evidence point to the existence of six stable genetic lineages or DTUs: TcI, TcIIa, TcIIb, TcIIc, TcIId, and TcIIe. Molecular dating suggests that *T. cruzi* is likely to have been an endemic infection of neotropical mammalian fauna for many millions of years. Here we have applied a panel of 49 polymorphic microsatellite markers developed from the online *T. cruzi* genome to document genetic diversity among 53 isolates belonging to TcIIc, a lineage so far recorded almost exclusively in silvatic transmission cycles but increasingly a potential source of human infection. These data are complemented by parallel analysis of sequence variation in a fragment of the glucose-6-phosphate isomerase gene. New isolates confirm that TcIIc is associated with terrestrial transmission cycles and armadillo reservoir hosts, and demonstrate that TcIIc is far more widespread than previously thought, with a distribution at least from Western Venezuela to the Argentine Chaco. We show that TcIIc is truly a discrete *T. cruzi* lineage, that it could have an ancient origin and that diversity occurs within the terrestrial niche independently of the host species. We also show that spatial structure among TcIIc isolates from its principal host, the armadillo *Dasypus novemcinctus*, is greater than that among TcI from *Didelphis* spp. opossums and link this observation to differences in ecology of their respective niches. Homozygosity in TcIIc populations and some linkage indices indicate the possibility of recombination but cannot yet be effectively discriminated from a high genome-wide frequency of gene conversion. Finally, we suggest that the derived TcIIc population genetic data have a vital role in determining the origin of the epidemiologically important hybrid lineages TcIId and TcIIe.

## Author Summary

*Trypanosoma cruzi*, the etiological agent of Chagas disease, infects over 10 million people in Latin America. Six major genetic lineages of the parasite have been identified with differential geographic distributions, ecological associations and epidemiological importance. With the advent of the *T. cruzi* genome sequence, it is possible to examine the micro-epidemiology of *T. cruzi* using high resolution genetic markers that assess diversity within these major types. Here we examine the genetic diversity of TcIIc, a poorly understood *T. cruzi* genetic lineage found predominantly among wild cycles of parasite transmission infecting terrestrial mammals and triatomine vectors, but also a potentially important emergent human disease agent. Amongst a number of findings, we show that TcIIc genetic diversity is comparable to other ancient *T. cruzi* lineages, highly spatially structured, and that a stringent co-evolutionary relationship with its principal reservoir host can be ruled out. Additionally, TcIIc is one of the two parents of hybrid lineages TcIId and TcIIe, which cause most of the Chagas disease that occurs in the Southern Cone of South America. The system we have developed will help to clarify the ecological circumstances around the emergence of these epidemiologically important hybrids, and perhaps help predict similar events in the future.

## Introduction

At least 10 million people are thought to carry the infectious agent of Chagas disease, *Trypanosoma cruzi*, which is considered to be responsible for ∼13,000 deaths annually (www.who.int, [1]). The disease is a vector-borne zoonosis and transmission in its wild transmission cycle is maintained by numerous species of mammal reservoir and over half of approximately 140 known species of haematophagous triatomine bug [2]. The geographical distribution of silvatic *T. cruzi* stretches from the Southern States of the USA to Southern Argentina. Domestic transmission is limited to Central and South America where domiciliated vector species occur. Human infection occurs primarily through mucosal or broken skin contact with contaminated triatomine faeces egested by the insect during feeding.

Consistent with an ancient association with South America [3] *T. cruzi* populations are highly diverse, with at least six stable discrete typing units (DTUs) reported: TcI, TcIIa, TcIIb, TcIIIc, TcIId, and TcIIe. Among these, TcI and TcIIb are the most divergent groups in molecular terms - estimates based on nuclear genes date their most recent common ancestor at 3–10 million years ago (MYA) [4]. The phylogenetic status of TcIIc and TcIIa is in full debate [5],[6]. Based on mosaic patterns of nucleotide diversity across nine nuclear genes, Westenberger *et al.*, (2005) proposed that both are the product of an early hybridisation event(s) between lineages TcI and TcIIb [6]. Others argue that TcIIc and TcIIa represent a single ancestral group in their own right [5], whereby these lineages share a characteristic mitochondrial genome distinct from both TcI and TcIIb. These hypotheses are not mutually exclusive and TcIIa and TcIIc are not easily distinguished based on mitochondrial sequences [4]. However, nuclear gene sequences consistently support their status as genetically separate clades [4], [6]–[8] and flow cytometric analysis across a panel of representative strains reveals that TcIIc and TcIIa genomes are divergent in terms of their absolute size [9]. The current tendency to group TcIIc and TCIIa as a single lineage is an oversimplification that may arise from Miles's original Z3 classification [10]. In fact Miles clearly defines an additional lineage in later publications – Z3/Z1 ASAT, which corresponds to TcIIc [11],[12]. Researchers attempting to classify a third major lineage, TcIII - corresponding loosely to TcIIc - almost entirely ignore TcIIa [5], as well the large divergence between North and South American TcIIa isolates [13]. By contrast, there is general consensus in the literature regarding the evolutionary origin of the two remaining lineages, TCIId and TCIIe. These are almost certainly hybrids and nucleotide sequence [4], microsatellite [5], and enzyme electrophoretic [14],[15] data show that the parents are TcIIc and TcIIb. In line with experimental data [16], maxicircle kinetoplast DNA inheritance in TcIId and TcIIe appears to have been uniparental [4],[5], and both retain a mitochondrial genome similar to that of TcIIc.

TcIIc is infrequently isolated from domestic transmission cycles. Sporadic reports of this lineage occur from domestic mammals in the Chaco region of Paraguay and Argentina as well as southern Brazil (*Canis familiaris* [17]–[19]) from humans in Brazil [19],[20] and from domestic triatomine bugs in Argentina and Peru (*T. infestans* [17] Miles M A, *unpublished*). In total, domestic TcIIc isolates make up only a handful of strains over >30 years of sampling. By contrast, other lineages - in particular TcI, TcIIb, TcIId and TcIIe - are common in humans, domestic mammals and vectors [21], TcI in northern South America and TcIIb, IId and IIe in the Southern Cone region.

Although rare to domestic transmission cycles, TcIIc occurs with relatively high frequency in the silvatic environment. We have shown that this DTU is almost exclusively associated with terrestrial transmission cycles and fossorial mammalian genera, including the Cingulata (armadillos) and terrestrial marsupials (*Monodelphis spp.* & *Philander frenata*) [19],[22]. Terrestrial rodents (*Dasyprocta spp.*, *Proechimys iheringi*, *Oryzomys spp.* and *Oxymyctereus sp.* [15],[19]) and Carnivora (*Conepatus spp.* [17]) have also been implicated. Among these hosts, the nine-banded armadillo, *Dasypus novemcinctus*, is probably the most important. In Paraguay [22] and Bolivia (Llewellyn *et al.*, *unpublished data*) prevalence of infection in this mammal is consistently 33%–57% across distinct geographic foci. Although *D. novemcinctus* does account for most of the TcIIc isolates sampled from mammalian reservoirs in the silvatic environment, it is unclear to what extent *D. novemcinctus* and TcIIc have shared a common evolutionary relationship. Trypanosomes rarely co-speciate with their hosts or vectors, instead ‘ecological-host fitting’ is thought to be the major driver behind parasite diversification [23] whereby parasite clades are associated with distinct vector/host cliques characteristic of a particular ecological niche. Thus far, few vector species have been incriminated in silvatic transmission of TcIIc. *Pantrongylus geniculatus* and *Triatoma rubrovaria*, both principally silvatic vectors and often, although not exclusively, associated with terrestrial ecotopes [24], as well as *Dasypus sp.* armadillos [2],[12],[25],[26], are both recorded with TcIIc infection [12],[19],[27]

The occurrence of TcIIc in domestic transmission cycles, albeit infrequently, implies a role as an agent of human disease. In addition, it is likely that TcIIc is under-reported from both domestic and silvatic transmission cycles because some typing methodologies fail to distinguish between TcIIa and TcIIc (e.g. [28]). Furthermore, TcIIc is one of the parents of the hybrid lineages TcIId and TcIIe [4], which are predominant agents of severe Chagas disease in the Gran Chaco and adjacent regions [21]. TcIIc therefore represents an important focus for study. As we have recently shown for TcI, an understanding of the dynamics of silvatic *T. cruzi* infection is a vital step before evaluating the nature of domestic parasite transmission [9]. For TcIIc, this rationale becomes important as human populations expand into previously undisturbed cycles of natural transmission and secondary vector species re-emerge from the silvatic environment after the eradication of major domestic species [29],[30]. With the aim of establishing the diversity of silvatic TcIIc, here we use 49 microsatellite loci, 12 newly identified in this study, in conjunction with sequence from the glucose-6-phosphate isomerase *(GPI)* gene to examine the population genetics of this lineage from foci across South America. We demonstrate that TcIIc populations are diverse, spatially structured and well established across different climatic regions in South America. By comparison to a newly available TcI microsatellite dataset, we are able to shed light on the ecological and evolutionary significance of our findings.

## Methods and Analyses

We assembled a panel of 53 *T. cruzi* samples belonging to TcIIc, including published, archived and original isolates (Table S2). Samples originate from five countries: Colombia, Brazil, Venezuela, Bolivia and Paraguay. Basic (DTU-level) genotyping of these strains was achieved through analysis of amplified fragment polymorphism in the D7 divergent domain of the 24Sα rRNA locus and restriction fragment length polymorphism in the heat shock protein 60 (*HSP60*) gene, as described previously [6],[31].

### Sequences

A c.1 kb fragment of the glucose-6-phosphate isomerase *(GPI)* gene was sequenced across a representative subset of 22 TcIIc isolates. Genbank accession numbers for the corresponding strains are included in Table S2. Amplification was achieved according to Gaunt *et al.*, (2003) using primers *gpi*.for (5′-CGC ACA CTG GCC CTA TTA TT) and *gpi*.rev (5′-TTC CAT TGC TTT CCA TGT CA) [16] in a final reaction volume of 25 ul containing containing 1× *Taq* polymerase reaction NH_4_^+^ buffer (Bioline, UK)), 2 mM MgCl_2_, 200 uM dNTPs; 25 pM of each primer, 1.25 units of *Taq* polymerase, and 35 ng of parasite DNA. The reaction cycle involved an initial denaturation step for five minutes at 94°C, followed by 28 amplification cycles (94°C for 30 seconds, 60°C for 30 seconds, 72°C for 30 seconds) and a final ten minute elongation step at 72°C. PCR products were prepared for sequencing with a BigDye® v3.1 sequencing kit (Applied Biosystems, UK), according to the manufacturer's instructions. In addition to forward and reverse external primers, one internal primer was also employed, *gpi.*1 (5′TGT GAA GCT TTG AAG CCT TT) [16]. Samples demonstrating two or more heterozygous sequence profiles at individual nucleotide sites were cloned individually using the pGEM T easyVector® system (Promega, UK) to derive sequence haplotypes. Owing to the reported occurrence (c.20% e.g [32]) of artefactual recombinant sequence haplotypes derived from *Taq* DNA polymerase template switching during PCR amplification, ten different clones were sequenced from each sample. Minority recombinant sequence artefacts were identified and excluded from the analysis.

### Phylogenetic analysis

**Figure 1:**
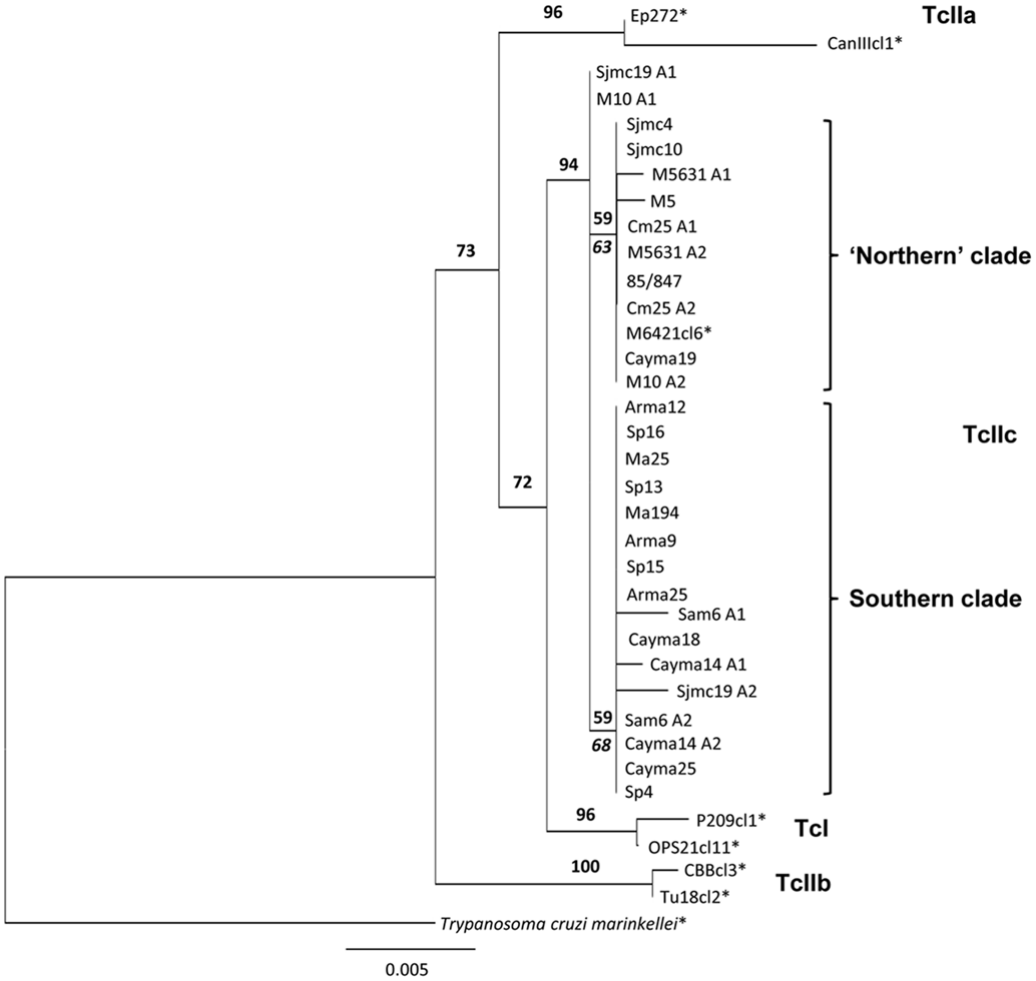
Phylogenetic relationships between 31 *Trypanosoma cruzi* isolates (25 TcIIc) based on a 980 bp fragment of the glucose-6-phosphate isomerase (*GPI*) gene. The tree was constructed under neighbour-joining using Kimura– 2–parameter distances. Bootstrap values are shown above major clades. Bootstrap values in italics below TcIIc intra-lineage clade branches are those generated after exclusion of outliers SJMC19 and M10. *Genbank sequences first published by Broutin *et al.*, 2006 [7]. A – allele or halpotype.

Analysis was undertaken of a 980 nucleotide sequence alignment of all experimentally derived haplotypes. Also included in this alignment were selected fragments available on Genbank from a recent study of *T. cruzi GPI* sequence diversity (AY484472–AY484478) [7]. Tree topology was defined using Kimura-2-parameter (k2p) distances and reconstructed through Neighbour-Joining (NJ) in the PHYLIP v3.67 software package [33]. A thousand bootstrapped datasets were generated in SEQBOOT, analysed using k2p distances, and the resultant NJ trees assessed for congruence in CONSENSE, all in PHYLIP v3.67 [33]. The resulting tree (Figure 1) was visualised and prepared for publication using FigTree v1.1.1 (http://tree.bio.ed.ac.uk/software/figtree/).

### Microsatellites

Repetitive motifs were extracted from the draft sequence of the *T. cruzi* genome available at www.genedb.org for analysis of all 53 TcIIc isolates. Four Mb of sequence, including at least 13 syntenous sequence fragments (SSFs), were scanned for di- and tri-nucleotide repeats using a pattern matching script (regular expression) written in *sed*. An extension of the algorithm was included to extract the up and downstream flanking regions of the microsatellite sequence (∼200 bp). Primer design was achieved in PRIMER3 [34]

Over 200 microsatellite loci were identified and screened against a representative subset of five TcIIc isolates. Forty-nine markers, polymorphic across the test group, were selected for further use, including two employed in previous studies [35]. Thirty-seven markers correspond to those we have employed in a recent study of TcI intra-lineage diversity [9]. Twelve are unique to this study. Primer codes, sequences and binding sites are listed in Supplementary Information (Table S3). After optimisation of annealing temperatures, the following reaction cycle was implemented across all loci: a denaturation step of 4 minutes at 95°C, followed by 30 amplification cycles (95°C for 20 seconds, 57°C for 20 seconds, 72°C for 20 seconds) and a final 20 minute elongation step at 72°C. Reaction conditions, with a final volume of 10 ul, were as follows: 1× ThermoPol Reaction Buffer (New England Biolabs (NEB), UK), 4 mM MgCl_2_, 34 uM dNTPs; 0.75 pmols of each primer, 1 unit of *Taq* polymerase (NEB, UK) and 1 ng of genomic DNA. Five fluorescent dyes were employed to label forward primers – 6-FAM and TET (Proligo, Germany) as well as NED, PET & VIC (Applied Biosystems, UK). Microsatellite allele sizes were determined using an automated capillary sequencer (AB3730, Applied Biosystems, UK) in conjunction with a fluorescently tagged size standard and were manually checked for errors. All isolates were typed “blind” to control for user bias.

### Microsatellite diversity analysis

**Figure 2:**
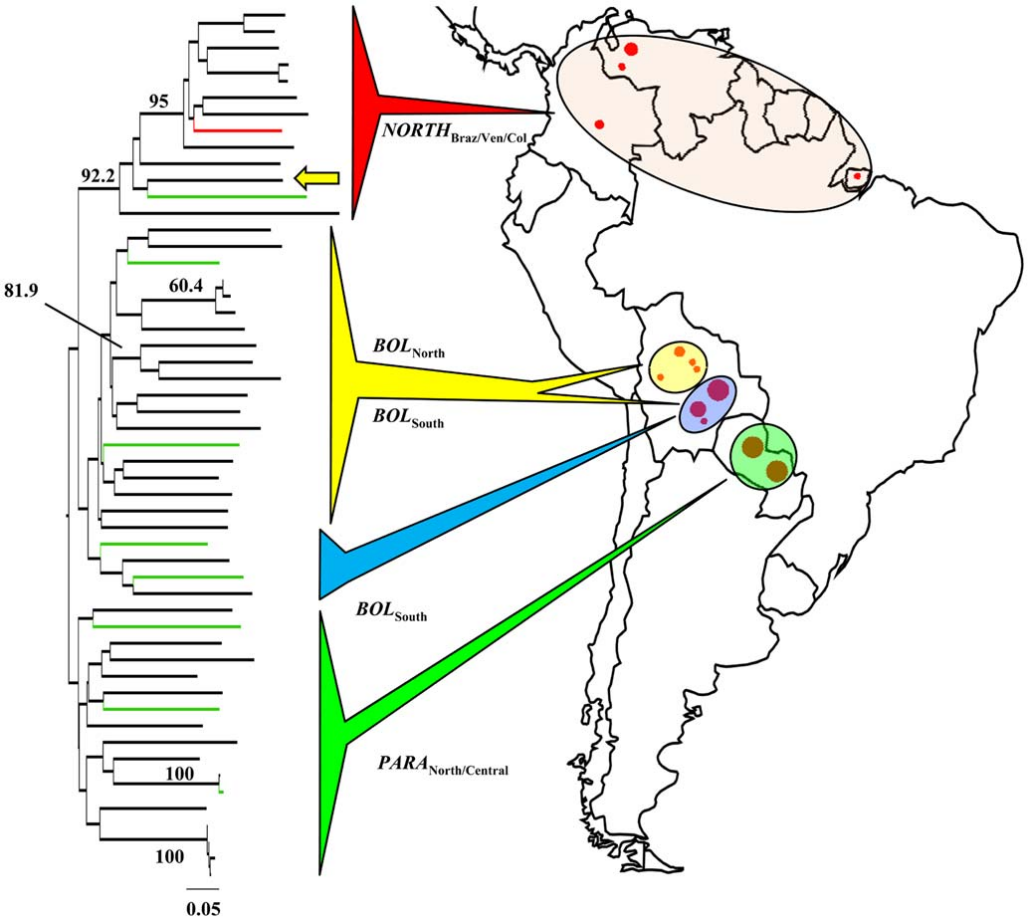
Unrooted neighbour-joining *D*_AS_ tree showing TcIIc population structure across South America. Based on the multilocus microsatellite profiles of 53 TcI isolates. *D*_AS_ values were calculated as the mean across 1000 random diploid re-samplings of the dataset to accommodate multi-allelic loci. The presence of more than two alleles per locus did not disrupt the delineation of major clades (>90% majority consensus support). *D*_AS_-based bootstrap values were calculated over 10,000 trees from 100 re-sampled datasets and those >60% are shown on major clades. Branch colour codes indicate strain origin. Black: *Dasypus* reservoir host species; Green: non-*Dasypus* reservoir host; Red: *Panstrongylus* vector species; Yellow arrow indicates Northern Bolivian outlier (SJMC19) assigned to *NORTH*_Braz/Ven/Col_ population. Closed red circle area is proportionate to sampling density. See text for details of population codes.

**Figure 3:**
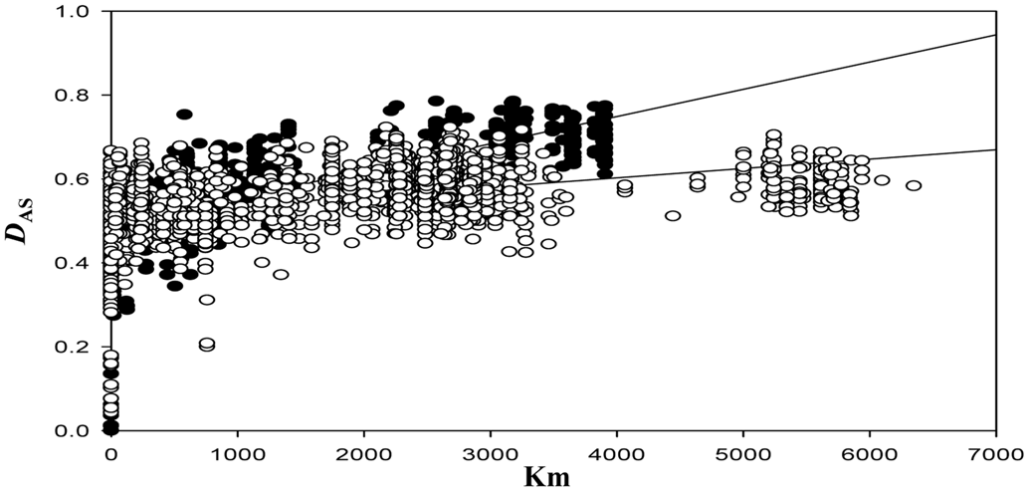
Isolation by distance among TcIIc and TcI from their respective major reservoir hosts compared. Graph shows the correlation between genetic (*D*_AS_) and geographic distance (km). Closed circles represent comparisons between TcIIc isolates from *Dasypus novemcinctus* and open circles TcI from *Didelphis marsupialis*. TcIIc isolates show significantly greater spatial structure. Regression statistics: TcIIc - *R*_XY_ = 0.658 p<0.001, regression gradient (RG) = 6.445×10^−5^+/−standard error (SE) = 2.401×10^−6^; TCI - *R*_XY_ = 0.429, p<0.001, RG = 2.234×10^−5^+/−SE 1.049×10^−6^.

Allelic richness estimates were calculated in FSTAT 2.9.3.2 [36] and corrected for sample size using Hurlbert's rarefaction method [37] in MolKin v3.0 [38] to obtain an unbiased measure of genetic polymorphism among those populations studied. Heterozygosity indices (Table 1) were estimated in ARLEQUIN 3.0 [39]. They include mean expected (under Hardy-Weinberg (HW) expectations) and observed heterozygosity over loci, as well as tests for deviation from HW equilibrium at the level of individual loci within populations. Pair-wise *F*_ST_ values were also estimated in ARLEQUIN v3.0 [39], and represent the proportion of variation accounted for by the sub-division between each population pair by comparison to the total level of variation across both populations. P-values for multiple tests were corrected using a sequential Bonferroni correction [40] to minimise potential Type 1 errors. A further statistic, *F*_IS_ was applied as an alternate measure of heterozygosity by assessing the level of identity of alleles within individuals compared to that between individuals where +1 represents all individuals homozygous for different alleles and −1 all individuals heterozygous for the same alleles. Mean *F*_IS_ values per SSF per population were calculated in FSTAT 2.9.3.2. to examine the genomic distribution of heterozygosity. Multilocus linkage disequilibrium, estimated by the Index of Association (I_A_), was calculated in MULTILOCUS 1.3b [41],[42] (Table 1) and tests for evidence of the non-random association of alleles across multiple loci. Genetic distances between isolates were evaluated in MICROSAT under an infinite alleles model of microsatellite evolution using *D*_AS_ (1-proportion of shared alleles at all loci / *n*) [43] (Figure 2). To accommodate multi-allelic loci, and asses their influence on the stability of the resulting tree, a script was written in Microsoft Visual Basic to make multiple random diploid re-samplings of each multilocus profile. Individual-level genetic distances were calculated as the mean across multiple re-sampled datasets. A single randomly sampled dataset was used for population-level analysis. A Mantel's test for matrix correspondence was executed in GENALEX 6 to compare pair-wise geographical (km) and genetic distance (*D*_AS_) [44] (Figure 3). Samples were assigned to populations on an *a priori* basis according to geography and transmission cycle. *D*_AS_ - defined sample clustering was also used to inform population identity, and obvious outliers assigned to the correct genetic group (Figure 2).

**Table 1:** Summary of key genetic parameters across four *Trypanosoma cruzi* IIc populations.

Population	N/G	A_r_	HO^a^	HE^a^	%HD^b^	%HE^c^	I_A_ d	p value
NORTH*_Braz/Ven/Col_*	13/13	2.73	0.327	0.578	15.9	0	6.72729	<0.001
BOL*_South_*	15/15	2.83	0.490	0.591	14.9	0	2.94509	<0.001
BOL*_North_*	8/8	2.82	0.514	0.650	4.8	0	1.20046	0.032
PARA*_North/Central_*	17/17	2.58	0.438	0.544	10.6	0	2.63024	<0.001

N = Number of isolates in population.

G = Number of multilocus genotypes per population.

A_r_ – Allelic richness (sample size corrected).

a Mean observed and expected heterozygosity across all loci.

b Proportion of loci showing a significant deficit in heterozygosity after a Bonferroni correction.

c Proportion of loci showing significant excess heterozygosity after a Bonferroni correction.

d Calculated in Multilocus v1.3 by comparison to a null distribution of 1000 randomizations drawn from the same dataset.

## Results

### Strains analysed

53 TcIIc isolates were assembled among which 33 are original to this study and were collected in Venezuela and Bolivia between 2005 and 2007. Venezuelan isolates were collected from the tropically forested foothills of the Cordillera Oriental to the west of the country around the town of Curbati, Barinas state (Sample prefix M & PARAMA). Three study sites in Bolivia fall across different ecological zones. The first, comparable in terms of ecotope but not elevation to the Venezuelan site was in low-lying Beni state (Sample prefix SJMO & SJM). The second was located in semi-arid Chiquitania dry forest c.60 km east of Santa Cruz de la Sierra (Sample prefix CAYMA) and the last in the arid Chaco region c.200 km south of Santa Cruz de la Sierra (Sample prefix MA & SAM). Isolates from Paraguay where collected by M. Yeo (MY) between 2001 and 2003 [22]. The northern study site at Campo Lorro lies in the arid Paraguayan Chaco (Sample prefix MA) and the southern site in semi-arid savannah in the central department of San Pedro (Sample prefix SP & ARMA). Four further historical isolates: M5361, CM17, CM25 & 85/847 are from North-Eastern Brazil, Eastern Colombia (CM) and Alto Beni (Bolivia) respectively. Among numerous mammal species sampled (>25 - Llewellyn *et al.*, unpublished; = 10 - MY [22]), most isolates originated from *D. novemcinctus*, including animals from Venezuela, Brazil, Colombia, Bolivia, and Paraguay. However, a number of secondary hosts were also present. In Colombia these included the terrestrial agouti, *D. fugilinosa*, in Bolivia armadillo genera *Euphractus sexcinctus* and *Chaetophractus vellorosus*, and in Paraguay *E. sexcinctus* and *C. vellorosus*, as well as the terrestrial marsupial *Monodelphis domestica*. A single isolate originates from a *Panstrongylus spp.* triatomine nymph found infesting a *D. novemcinctus* burrow at Curbati, Venezuela.

### Phylogenetic analyses

The tree resulting from sequence analyses is shown in Figure 1. Nine *GPI* sequence haplotypes were resolved among the 25 isolates analysed, and nine variable sites identified - equating to ∼0.9% sequence diversity within the TcIIc group. TcIIc emerged as a moderately well supported sister group to TcI (72% bootstrap support) and clearly distinct to those TcIIa strains included in the analysis. Among the TcIIc group, some correlation with geography was observed. The following, weakly supported (>50%), clades were apparent: a ‘northern’ group, corresponding to isolates from Brazil, Venezuela, Colombia and Bolivia; and a southern group, corresponding exclusively to isolates from Paraguay and Bolivia. This subdivision corresponds to two fixed single nucleotide polymorphisms between the two groups. One sequence haplotype, (sjmc19_h1 and m10_hap1) fell as an outlier, and could not be assigned to either group. Removal of these isolates from the analysis improved resolution of the subdivision within the TcIIc group. Phylogenetic clustering occurred independently of host species.

### Microsatellite analysis

A final dataset of 4,585 alleles (excluding missing data) was subjected to analysis. Most strains presented one or two alleles at each locus. Multiple (≥3) alleles were observed at a small proportion of loci (0.45%), only among uncloned strains, and indicate the possible presence of polyclonal infections in reservoir hosts sampled. Four populations were defined: Venezuela, Colombia and Brazil (*NORTH*_Braz/Ven/Col_); Northern Bolivia (*BOL*_North_), Southern Bolivia (*BOL*_South_) and Paraguay (*PARA*_North/Central_).

### TcIIc genetic diversity measures

Measures of sample size-corrected genetic diversity (Allelic richness (A_r_), were relatively homogeneous across all populations (Ar = 2.58–2.83, Table 1), and no support for a specific correlation between genetic diversity and geographic origin was identified. Diversity indices across all TcIIc populations were equivalent to those observed in lowland silvatic TcI populations (A_r_ = 2.23–2.34) [9]. Identical TcIIc multilocus genotypes (MLGs) were not observed, and clone correction (removal of identical MLGs) unnecessary in the calculation of parameters from the current dataset.

### Pair-wise measures of genetic distance

Isolate clustering based on pair-wise *D*_AS_ values (Figure 2) revealed clades broadly defined by geographical origin. Strong bootstrap support (92.2%) was found for a division between isolates from Northern and Southern South America. SJMC19 (Table S2), isolated from *D. novemcinctus* in *BOL*_North_, and defined by *GPI* sequence data as an outlier on the basis of one halplotype, represents a possible migrant and fell within the Northern cluster on the basis of microsatellite variation. As such it was assigned to *NORTH*_Braz/Ven/Col_ for population level analyses. Consistent with physical proximity, no bootstrap support was apparent between clades from Bolivia and Paraguay. As with *GPI* sequence data, partitioning of isolates by host was not apparent in this dataset. A tree based on pair-wise distances was also constructed under a step-wise model of microsatellite mutation (δμ^2^ [45]) and bootstrapped using the same methodology as that in Figure 2. Overall the result was poor by comparison to the *D*_AS_ derived topology. The bootstrap value for the major division between northern and southern South America, for example, was c. 3%.

**Figure 4:**
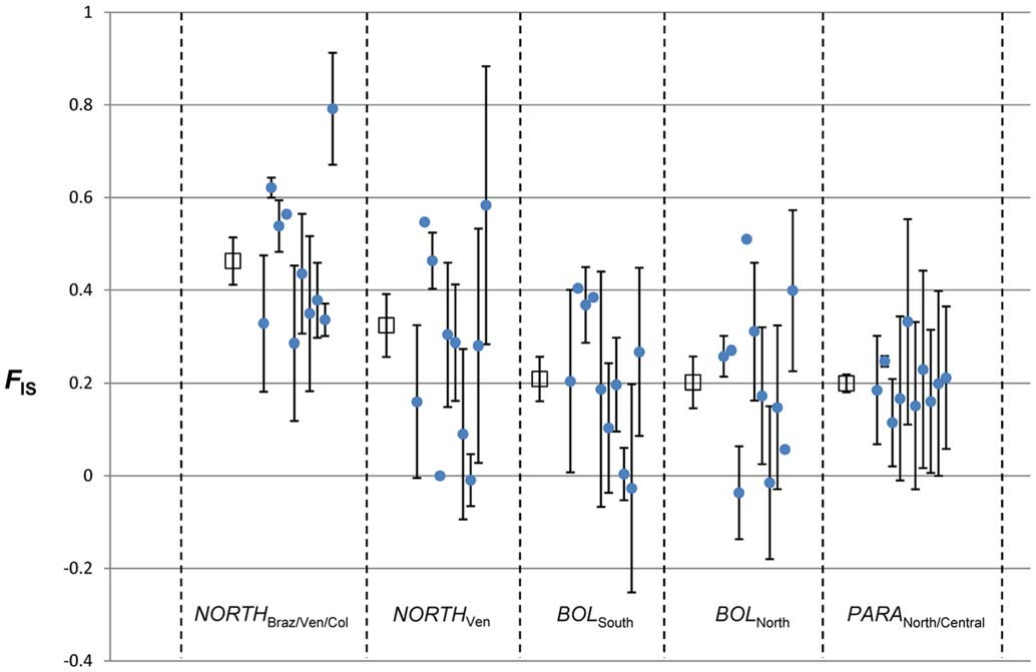
Mean *F*_IS_ values per syntenous sequence fragment (SSF) across five TcIIc populations. Error bars represent standard error about the mean. Open squares represent mean *F*_IS_ across all SSFs in each population. Closed circles represent mean *F*_IS_ per SSF. Missing error bars correspond to SSFs containing only a single variable locus. *NORTH*_Ven_ was defined from *NORTH*_Braz/Ven/Col_ to examine the effect of excluding outlying isolates in order to minimise intrapopulation subdivision.

The extent of spatial structuring among isolates was tested by examining the relationship between genetic (*D*_AS_) and geographical distance (km). Strongly significant (*R*_XY_ = 0.687, p<0.001) isolation by distance was apparent across all TcIIc isolates. To facilitate a direct comparison between the spatial dynamics of two distinct *T. cruzi* major genotypes with their principal reservoir species, TcIIc isolates drawn exclusively from *D. novemcinctus* were compared with a larger dataset of TcI isolates from *Didelphis spp.* (*D. marsupialis* and *D. albiventris*) [9] (Figure 4). The following conclusions can be drawn: 1) Both *D. novemcinctus* TcIIc isolates and *D. marsupialis* TcI isolates show significant spatial structure (TcIIc - *R*_XY_ = 0.658, p<0.001; TcI - *R*_XY_ = 0.429, p<0.001). Furthermore, the standard error (SE) about the regression gradient (RG) for each does not encompass zero, confirming this result. 2) TcIIc isolates from *D. novemcinctus* show greater spatial structure than TcI from *D. marsupialis* as the RG of the former (TcIIc - RG = 6.445×10^−5^+/−SE 2.401×10^−6^) is greater than the latter (TcI -RG = 2.234×10^−5^+/−SE 1.049×10^−6^) and the respective error bars do not overlap. Importantly TcIIc and TcI isolates from their respective principal host species were sampled across approximately the same geographical range, validating a direct comparison between the two (Figure 2, [9]).

### Heterozygosity measures

Heterozygous deficiency with respect to HW expectations was a consistent phenomenon across all population examined (Table 1). This effect was most pronounced in *NORTH*_Braz/Ven/Col_. To explore the genomic distribution of homozygosity, mean *F*_IS_ values were calculated from each SSF (as defined by the online CL Brener genome – www.tigr.com) containing ≥2 microsatellite loci. The results of this analysis are displayed in Figure 3 and suggest that homozygosity is fairly evenly distributed across the SSFs studied and by extension homozygosity is likely to be a genome-wide phenomenon. Notably, when Brazilian, Colombian and Bolivian isolates were excluded from *NORTH*_Braz/Ven/Col_, a marked reduction in *F*_IS_ was observed (Figure 3). Thus, to an extent, high levels of homozygosity within this population may be partially attributable to intra-population subdivision (Wahlund effect [46] *sensu lato* as in [47]).

### Population subdivision

Significant pair-wise inter-population subdivision (*F*_ST_) (p<0.004) after a sequential Bonferroni correction (Table S1) indicates that all populations studied are fairly discrete in population genetic terms, and values broadly correspond to the geographical distances involved (e.g. lowest subdivision is observed between populations closest geographically - *BOL*_North_ and *BOL*_South_ (*F*_ST_ = 0.051)). In support of differential levels of spatial structuring between TcIIc and TcI as summarised earlier, gene flow between a silvatic TcI population from *BOL*_North_ and populations from lowland Venezuela and North-Eastern Brazil was higher than that observed from the TcIIc dataset [9]. However, a possible confounder was the anomalous position of isolate SJMC19, which clustered alongside isolates from *NORTH*_Braz/Ven/Col_. In this case subdivision between *BOL*_North_ and *NORTH*_Braz/Ven/Col_ (*F*_ST_ = 0.284) is likely to have been marginally overestimated.

### Linkage disequilibrium in TcIIc

Strongly significant (p<0.001, Table 1) linkage disequilibrium (measured using the Index of Association (I_A_)) was detected in all populations except *BOL*_North_ where only marginal significance was observed (p = 0.032, Table 1). Predominantly clonal parasite propagation is thus supported by the non random association of alleles at different loci in most populations. However, given that the I_A_ is a highly conservative measure, some level of recombination cannot be ruled out in any population, especially *BOL*_North_.

## Discussion

The widespread spatial distribution and genetic diversity of the TcIIc isolates studied here point to an possible ancient origin for this DTU and certainly a long-term association with terrestrial transmission cycles. Historically, most TcIIc isolates have originated from the Southern Cone region of South America [19],[22]. We can now confirm that TcIIc occurs as far north as Western Venezuela, and by implication throughout the continent. Levels of genetic diversity among populations studied are comparable to those observed in arboreal silvatic TcI from lowland moist forest ecotopes [9]. Indeed there is no evidence from the current dataset to suggest that TcIIc is any ‘younger’ than TcI in evolutionary terms, although microsatellites may be a poor estimator of ancient evolutionary events. Nonetheless, the divergent TcIIc mitochondrial genome (i.e. kinetoplast maxicircle) does suggest an ancient origin for this lineage [4],[5] and lends support to our data. Also, observed heterozygous deficiency is not superficially consistent with a hybrid origin for TcIIc [6]. Again, however, microsatellites are an imperfect tool for detecting ancient hybrid signatures. Informative variation will be lost rapidly via mutation and/or gene conversion.

Genetic diversity was surprisingly homogenous across the populations studied, an observation interesting in the context of the major host species examined. Molecular dating of the long-nosed armadillos, the Dasypodini (which includes *Dasypus spp.*) suggests an early emergence for this group (c.40 MYA), if not for the species *D. novemcinctus* itself, which is likely to have emerged later [48]. The ancestors of extant *Dasypus* species were presumably widespread in the tropical-temperate forest environments that predominated throughout South America around this time [49]. The emergence of the extant Euphractinae (which include *Chaetophractus* and *Euphractus spp*) is thought to have occurred very recently (c.5 MYA) in response to climatic cooling and the formation of the arid southern Chaco and Pampas ecotopes [48]. Diversity estimates from our data reject a recent radiation of TcIIc into Paraguay and Southern Bolivia in conjunction with the emergence of Euphractinae hosts. It seems instead that residual populations of *Dasypus spp.* have maintained TcIIc transmission in dryer areas, and indeed these mammals demonstrate a much higher infection rate in Southern Bolivia (Llewellyn *et al.*, unpublished data) and Northern Paraguay [22] than other dry-adapted armadillo genera, despite being less abundant. This observation could be related to the ease with which the burrows of different armadillo genera are infested with triatomines. Our field observations suggest that *Tolypeutes matacus* (rarely, if ever infected - Llewellyn *et al.*, unpublished, [22]) does not dig burrows; *E. sexcinctus* and *Chaetophractus spp* (infrequently infected Llewellyn *et al.*, unpublished, [22]) dig very deep burrows; whereas *D. novemcinctus* burrows are shallower, subject to repeated use by the same individual and provide an easily accessible long-term refuge for triatomines. Nonetheless, triatomines do transmit TcIIc to other terrestrial genera and secondary hosts must have fairly frequent contact with this DTU as *D. novemcinctus* and *non-D. novemcinctus* isolates are not clearly distinguishable at discrete foci. TcIIc is thus eclectic in terms of host in terrestrial transmission cycles, as expected under a model of ‘ecological host-fitting’ [23]. It follows that a stringent co-evolutionary relationship with *D. novemcinctus* can be ruled out in the context of the current dataset, and, in the context recent data from Brazil, with other known hosts of TcIIc [19]. Interestingly, a new isolate from a *Panstrongylus spp.* nymph in Barinas, Venezuela (M3-CU), recovered from the burrow of *D. novemcinctus* corroborates earlier reports of TcIIc from this vector genus in North-Eastern Brazil [12], and provides more support for ‘divergence by niche’ in *T. cruzi* silvatic populations [22].

On the basis of microsatellite diversity, and concordant with a related study in Brazil [19], TcIIc is highly spatially structured across South America. This observation corresponds with the general epidemiology of silvatic disease transmission, where endemic parasite populations at distinct foci exchange little genetic content in the absence of rapid and long distance host or vector dispersal. SJMC19, a strain isolated from *D. novemcinctus* in Northern Bolivia, is an exception being apparently a northern migrant. However, the grouping of SJMC19 with isolates from *NORTH*_Braz/Ven/Col_ could be an artefact of poor sample coverage from Western Brazil and warrants more intensive sampling from this region. A statistical comparison between TcI and TcIIc isolates from their major reservoirs (*D. marsupialis* and *D. novemcinctus* respectively) reveals greater spatial structuring among the latter. This perhaps relates to the larger home range of *D. marsupialis* as compared to *D. novemcinctus* [50], but also to the greater number of secondary hosts involved in TcI transmission [22], if historical records are broadly representative of the relative abundance of the two lineages among mammalian genera. *GPI* sequence data provide a more confused pattern of spatial diversification, where, among the 24 TcIIc strains analysed, the North-South divide is less pronounced. A single nuclear locus, especially from a relatively conserved sequence class, is clearly insufficient to address a population genetic question. However, sequence data (Figure 1) do corroborate the anomalous status of SJMC19, and two highly divergent haplotypes are evident in this sample, one identical to a Venezuelan haplotype (itself an outlier (M10 A1)), and the other occurring alongside haplotypes from Paraguay, Central and Southern Bolivia, potentially consistent with recombination and worthy of further study.

A common feature between both TcI [9] and TcIIc isolates, and consistent within *T. cruzi* as a whole [4],[6],[35], with the exception of hybrids TcIId and TcIIe, is an apparent lack of heterozygosity as compared to Hardy-Weinberg expectations. Heterozygous deficiency is also incongruent with extreme models of long term clonal evolution in diploids, where haplotypes are expected to become increasingly divergent over time in the absence of recombination [47], [51]–[53]. Excess homozygosity in sexual populations, assuming strict neutrality, zero allele drop out and discounting Wahlund effects, is normally indicative of inbreeding [54]. Heterozygosity in predominantly clonal diploids such as *T. cruzi* can theoretically be reduced by several processes including gene conversion and occasional recombination (both out-crossing and selfing events), but distinguishing between these processes is challenging.

As in our recent study of TcI microsatellite diversity [9], we can show that homozygosity in *T. cruzi* is genomically diffuse. This suggests that infrequent, localised (e.g. whole chromosomes or chromosome fragments) gene conversion events can, therefore, be ruled out in the context of those SSFs we examined. A recent population genetic study of a related trypanosomatid (*Leishmania braziliensis*), previously thought to be clonal, partially attributes excess homozygosity to endogamic recombination [55]. We found no concrete evidence for sexuality within the TcIIc populations studied, but some level of recombination cannot be ruled out, especially in *BOL*_North_, where only marginal significance could be attributed to the Index of Assocation (multilocus linkage disequilibrium [42]), which is considered a conservative measure of clonality [47]. Two important issues must be considered when attempting to distinguish between the various non-exclusive sources of homozygosity in a predominantly clonal diploid: 1) It seems illogical to correct for Wahlund effects using population assignment programs that explicitly rely upon Hardy-Weinberg assumptions with the aim of demonstrating endogamic sexuality [55],[56] – this argument is circular. 2) In order to discount gene conversion by disproving a negative relationship between allele size differences in heterozygotes and the number of heterozygotes across samples, one must assume a purely stepwise model of microsatellite mutation [55], without significant frequencies of back mutation or homoplasy. In our analysis we were able provide some evidence of a Wahlund effect *sensu lato* by manual exclusion of outlying samples from *NORTH*_Braz/Ven/Col_. However, we were unable to discount gene conversion as a source of homozygosity as the step-wise model we applied to our data seemed to give poor results by comparison to *D*_AS_. Null loci could contribute to the homozygosity observed in our dataset, however, primers were designed against the CL-Brener genome, of which one haplotype belongs to a TcIIc parent, and we do not therefore expect major sequence divergence between the microsatellite flanking regions of this and our TcIIc isolates. We would therefore cautiously suggest that both a high frequency of gene conversion acting across the genome as well as hybridisation involving fusion of highly similar or identical individuals could have played a role in generating the observed diversity but we are unable to discriminate between the two processes with any confidence. Additionally we suggest that the latter process would be best demonstrated experimentally in TcIIc, as it has been in TcI [16], before drawing direct conclusions from variation at microsatellite loci, about which the mutational mechanism is still poorly understood.

Whether or not recombination is an important factor, we believe it is a valid interpretation of our data, and that of others [4], [6]–[8], that TcIIc represents an ancient, discrete and diverse *T. cruzi* lineage with well defined ecological associations and a continental distribution among silvatic cycles of parasite transmission. Despite a strong association with *D. novemcinctus*, TcIIc is eclectic within the terrestrial ecotope and parasite diversification occurs independently of host species. We can also confirm that the dispersal of this lineage between foci of transmission occurs at a significantly lower rate than that of TcI, a phenomenon that may be partly explained by differential primary host dynamics. While we recognize that the inclusion of TcIIc within the ‘TcII’ group makes increasingly little taxonomic sense, it also makes no more or less sense than the inclusion of any of the other TcII groups under the same heading. *T. cruzi* is certainly overdue a taxonomic overhaul, but, until further clarification - which must include multilocus analysis of a larger number of strains, especially from TcIIa and TcIIb – we believe that the DTU definition [57], which implies monophyly within clades but makes no assumptions about the evolutionary relationship between clades, is currently the ‘least wrong’ in terms of *T. cruzi* population structure.

Interestingly, TcIIc appears to be absent from the USA on the basis of the current literature, and among the *D. novemcinctus* so far sampled only TcI (N = 2) and TcIIa (N = 1) have been identified [58]. *D. novemcinctus* is widespread in the Southern USA, and if overall *T. cruzi* prevalence is comparable to that we have identified in South America [22] (Llewellyn *et al.*, unpublished) this species has been heavily under-sampled. In terms of human transmission, our dataset and analytical methodology will be applicable in pinpointing the geographical and/or ecological origin of the predominantly domestic *T. cruzi* strains TcIId and TcIIe. Westenberger *et al.*, 2006 [59] provide evidence from the composition of 5S rRNA arrays that the TcIIb ancestor of TcIId and TcIIe lies within the western portion of the Southern Cone of South America. Our microsatellite panel can now provide information with regards to the TcIIc ancestor, as well as more fine scale determination of the likely TcIIb ancestor, so long as adequate samples are available. In doing so it may be possible to clarify the ecological circumstances around the emergence of these epidemiologically important hybrids, and perhaps help predict similar events in the future.

## Supporting Information

Table S1 Pair-wise estimates of *F*_ST_ between four TcIIc populations.
(0.03 MB DOC)

Table S2 *Trypanosoma cruzi* strains analysed in this study.
(0.11 MB DOC)

Table S3 Microsatellite loci used in this study.
(0.15 MB DOC)
