# Supplementary material for: Trypanosoma cruzi IIc: Phylogenetic and Phylogeographic Insights from Sequence and Microsatellite Analysis and Potential Impact on Emergent Chagas Disease
Source: PLoS Negl Trop Dis. 2009 Sep 1;3(9):e510. doi: 10.1371/journal.pntd.0000510 (PMC2727949; doi:10.1371/journal.pntd.0000510)
Supplement: Table S1 — Pair-wise estimates of F ST between four TcIIc populations. (0.03 MB DOC) [file pntd.0000510.s001.doc]

Table S1 Pair-wise estimates of *F*_ST_ between four TcIIc populations

| NORTH*_Braz/Ven/Col_* | * | *0.000* | *0.000* | *0.000* |
| --- | --- | --- | --- | --- |
| BOL*_South_* | 0.284 | * | *0.004* | *0.000* |
| BOL*_North_* | 0.282 | 0.051 | * | *0.000* |
| PARA*_North/Central_* | 0.334 | 0.166 | 0.175 | * |

*F*_ST_ estimates of inter-population differentiation between four TCIIc subpopulations based on microsatellite data. Italics indicate p-values generated from 1000 random permutations leading to a value lager than or equal to that observed. All values remain significant after Bonferroni correction**.**
