# Supplementary material for: Trypanosoma cruzi IIc: Phylogenetic and Phylogeographic Insights from Sequence and Microsatellite Analysis and Potential Impact on Emergent Chagas Disease
Source: PLoS Negl Trop Dis. 2009 Sep 1;3(9):e510. doi: 10.1371/journal.pntd.0000510 (PMC2727949; doi:10.1371/journal.pntd.0000510)
Supplement: Table S2 — Trypanosoma cruzi strains analysed in this study. (0.11 MB DOC) [file pntd.0000510.s002.doc]

Table S2 – *Trypanosoma cruzi* IIc strains analysed in this study.

| **Host** | **Code** | **Accession**  **number** | **Date** | **Locality** | **Latitude^b^** | **Longitude^b^** | **Population** | **Source^e^** |
| --- | --- | --- | --- | --- | --- | --- | --- | --- |
|  |  |  |  |  |  |  |  |  |
| *Dasypus novemcinctus* | **85/847** | GQ380661 | Unknown | Alto Beni, Bolivia | -15.5 | -67.5 | BOL*_North_* | IRD |
| *Dasypus novemcinctus* | SJMC10 | GQ380662 | 13.09.04 | Beni, Bolivia | -14.81 | -64.6 | BOL*_North_* | TS |
| *Dasypus novemcinctus* | SJMC4 | GQ380663 | 09.09.04 | Beni, Bolivia | -14.81 | -64.6 | BOL*_North_* | TS |
| *Dasypus novemcinctus* | **SJMO18** |  | 21.06.07 | Beni, Bolivia | -15.12 | -64.32 | BOL*_North_* | TS |
| *Dasypus novemcinctus* | **SMA18** |  | 09.06.07 | Beni, Bolivia | -14.13 | -65.36 | BOL*_North_* | TS |
| *Dasypus novemcinctus* | **SMA19** |  | 09.06.07 | Beni, Bolivia | -14.13 | -65.36 | BOL*_North_* | TS |
| *Dasypus novemcinctus* | **SMA8XE** |  | 08.06.07 | Beni, Bolivia | -14.13 | -65.36 | BOL*_North_* | TS |
| *Dasypus novemcinctus* | **SMA9** |  | 08.06.07 | Beni, Bolivia | -14.13 | -65.36 | BOL*_North_* | TS |
| *Dasypus novemcinctus* | CAYMA13 |  | 17.01.05 | Santa Cruz, Bolivia | -17.5 | -61.5 | BOL*_South_* | TS |
| *Dasypus novemcinctus* | CAYMA14 | GQ380664/  GQ380665 | 14.01.05 | Santa Cruz, Bolivia | -17.5 | -61.5 | BOL*_South_* | TS |
| *Dasypus novemcinctus* | CAYMA17 |  | 17.01.05 | Santa Cruz, Bolivia | -17.5 | -61.5 | BOL*_South_* | TS |
| *Dasypus novemcinctus* | CAYMA18 | GQ380666 | 17.01.05 | Santa Cruz, Bolivia | -17.5 | -61.5 | BOL*_South_* | TS |
| *Dasypus novemcinctus* | CAYMA19 | GQ380667 | 17.01.05 | Santa Cruz, Bolivia | -17.5 | -61.5 | BOL*_South_* | TS |
| *Dasypus novemcinctus* | **CAYMA21** |  | 17.01.05 | Santa Cruz, Bolivia | -17.5 | -61.5 | BOL*_South_* | TS |
| *Euphractus sexcinctus* | CAYMA25 | GQ380668 | 17.01.05 | Santa Cruz, Bolivia | -17.5 | -61.5 | BOL*_South_* | TS |
| *Euphractus sexcinctus* | **CAYMA3** |  | 06.01.05 | Santa Cruz, Bolivia | -17.5 | -61.5 | BOL*_South_* | TS |
| *Chaetophractus vellorosus* | MA202 |  | 04.08.05 | Santa Cruz, Bolivia | -19.21 | -63.43 | BOL*_South_* | TS/NA |
| *Dasypus novemcinctus* | MA204 |  | 05.08.05 | Santa Cruz, Bolivia | -19.21 | -63.43 | BOL*_South_* | TS/NA |
| *Dasypus novemcinctus* | MA215 |  | 12.08.05 | Santa Cruz, Bolivia | -19.21 | -63.43 | BOL*_South_* | TS/NA |
| *Dasypus novemcinctus* | MA219 |  | 13.08.05 | Santa Cruz, Bolivia | -19.21 | -63.43 | BOL*_South_* | TS/NA |
| *Dasypus novemcinctus* | MA220 |  | 13.08.05 | Santa Cruz, Bolivia | -19.21 | -63.43 | BOL*_South_* | TS/NA |
| *Euphractus sexcinctus* | MA222 |  | 13.08.05 | Santa Cruz, Bolivia | -19.21 | -63.43 | BOL*_South_* | TS/NA |
| *Dasypus novemcinctus* | SAM6 | GQ380669/  GQ380670 | 21.11.04 | Santa Cruz, Bolivia | -20.02 | -63.02 | BOL*_South_* | TS |
| *Dasypus spp* | **CM 17** |  | 1982 | Carimaga, Colombia | 3.3 | -73 | NORTH*_Braz/Ven/Col_* | IRD |
| *Dasyprocta fugilinosa* | **CM 25** | GQ380671/  GQ380672 | 1982 | Carimaga, Colombia | 3.3 | -73 | NORTH*_Braz/Ven/Col_* | IRD |
| *Panstrongylus sp* | M3-CU |  | 03.05.04 | Barinas, Venezuela | 8.48 | -70.73 | NORTH*_Braz/Ven/Col_* | TS |
| *Dasypus novemcinctus* | M10 | GQ380673  GQ380674 | 17.05.04 | Barinas, Venezuela | 8.48 | -70.73 | NORTH*_Braz/Ven/Col_* | TS |
| *Dasypus novemcinctus* | M5 | GQ380675 | 09.05.04 | Barinas, Venezuela | 8.48 | -70.73 | NORTH*_Braz/Ven/Col_* | TS |
| *Dasypus novemcinctus* | **M5631** | GQ380676/  GQ380677 | c.1980 | Marajo. Brazil | -1 | -49.5 | NORTH*_Braz/Ven/Col_* | LSHTM |
| *Dasypus novemcinctus* | M6 |  | 09.05.04 | Barinas, Venezuela | 8.48 | -70.73 | NORTH*_Braz/Ven/Col_* | TS |
| *Dasypus novemcinctus* | M8 |  | 15.05.04 | Barinas, Venezuela | 7.5 | -71.23 | NORTH*_Braz/Ven/Col_* | TS |
| *Dasypus novemcinctus* | PARAMA26 |  | 06.08.05 | Barinas, Venezuela | 8.43 | -70.55 | NORTH*_Braz/Ven/Col_* | TS |
| *Dasypus novemcinctus* | PARAMA25 |  | 06.08.05 | Barinas, Venezuela | 8.43 | -70.55 | NORTH*_Braz/Ven/Col_* | TS |
| *Dasypus novemcinctus* | PARAMA34 |  | 06.08.05 | Barinas, Venezuela | 8.43 | -70.55 | NORTH*_Braz/Ven/Col_* | TS |
| *Dasypus novemcinctus* | PARAMA6 |  | 06.08.05 | Barinas, Venezuela | 8.43 | -70.55 | NORTH*_Braz/Ven/Col_* | TS |
| *Dasypus novemcinctus* | SJMC19 | GQ380678/  GQ380679 | 09.09.04 | Beni, Bolivia | -14.81 | -64.6 | NORTH*_Braz/Ven/Col_* | TS |
| *Dasypus novemcinctus* | ARMA12 | GQ380680 | 2001 | Campo Lorro, Paraguay | -22.33 | -58.93 | PARA*_North/Central_* | MY |
| *Dasypus novemcinctus* | **ARMA13** |  | 2001 | Campo Lorro, Paraguay | -22.33 | -58.93 | PARA*_North/Central_* | MY |
| *Dasypus novemcinctus* | **ARMA18** |  | 2001 | Campo Lorro, Paraguay | -22.33 | -58.93 | PARA*_North/Central_* | MY |

| **Host** | **Code** | **Accession number** | **Date** | **Locality** | **Latitude^a^** | **Longitude^a^** | **Population^b^** | **Source^c^** |
| --- | --- | --- | --- | --- | --- | --- | --- | --- |
|  |  |  |  |  |  |  |  |  |
| *Dasypus novemcinctus* | ARMA24 |  | 2001 | San Pedro, Paraguay | -24 | -57 | PARA*_North/Central_* | MY |
| *Dasypus novemcinctus* | ARMA25 | GQ380681 | 2001 | San Pedro, Paraguay | -24 | -57 | PARA*_North/Central_* | MY |
| *Dasypus novemcinctus* | ARMA26 |  | 2001 | San Pedro, Paraguay | -24 | -57 | PARA*_North/Central_* | MY |
| *Dasypus novemcinctus* | ARMA27 |  | 2001 | San Pedro, Paraguay | -24 | -57 | PARA*_North/Central_* | MY |
| *Dasypus novemcinctus* | ARMA9 | GQ380682 | 2001 | Campo Lorro, Paraguay | -22.33 | -58.93 | PARA*_North/Central_* | MY |
| *Chaetophractus vellorosus* | MA194 | GQ380683 | 27.01.05 | Campo Lorro, Paraguay | -22.33 | -58.93 | PARA*_North/Central_* | TS/NA |
| *Euphractus sexcinctus* | MA25* | GQ380684 | 2003 | Campo Lorro, Paraguay | -22.33 | -58.93 | PARA*_North/Central_* | MY |
| *Euphractus sexcinctus* | MA25X* |  | 2003 | Campo Lorro, Paraguay | -22.33 | -58.93 | PARA*_North/Central_* | MY |
| *Dasypus novemcinctus* | MA87 |  | 2003 | Campo Lorro, Paraguay | -22.33 | -58.93 | PARA*_North/Central_* | MY |
| *Dasypus novemcinctus* | SP13 | GQ380685 | 2003 | San Pedro, Paraguay | -24 | -57 | PARA*_North/Central_* | MY |
| *Dasypus novemcinctus* | SP14 |  | 2003 | San Pedro, Paraguay | -24 | -57 | PARA*_North/Central_* | MY |
| *Dasypus novemcinctus* | SP15 | GQ380686 | 2003 | San Pedro, Paraguay | -24 | -57 | PARA*_North/Central_* | MY |
| *Dasypus novemcinctus* | SP16 | GQ380687 | 2003 | San Pedro, Paraguay | -24 | -57 | PARA*_North/Central_* | MY |
| *Monodelphis domestica* | SP4 | GQ380688 | 2003 | San Pedro, Paraguay | -24 | -57 | PARA*_North/Central_* | MY |
|  |  |  |  |  |  |  |  |  |

Samples in bold were biologically cloned during this study.

*Isolated by MY from the same mammal using two different techniques: MA25 - direct inoculation from whole blood; MA25X – xenodiagnostic isolation.

^a^ Decimal degrees.

^b^ Population – see main text for details.

^c^ **IRD** – Institut de Recherche pour le Developpement, Montpellier, France, courtesy of C. Barnabe. **LSHTM** – held at LSHTM, London. **TS** – Isolated during this study by M. Llewellyn. **TS/NA –** Isolated during this study in collaboration with Nidia Acosta (LSHTM). **MY** – Isolated by M. Yeo, LSHTM.
