# Supplementary material for: Trypanosoma cruzi IIc: Phylogenetic and Phylogeographic Insights from Sequence and Microsatellite Analysis and Potential Impact on Emergent Chagas Disease
Source: PLoS Negl Trop Dis. 2009 Sep 1;3(9):e510. doi: 10.1371/journal.pntd.0000510 (PMC2727949; doi:10.1371/journal.pntd.0000510)
Supplement: Table S3 — Microsatellite loci used in this study. (0.15 MB DOC) [file pntd.0000510.s003.doc]

Table S3 – Microsatellite loci used in this study.

| **Primer code** | **Contig ID^a^** | **Position^b^** | **Repeat type^c^** | **Forward/ Reverse primer (5'-3')** |
| --- | --- | --- | --- | --- |
|  |  |  |  |  |
| 6529(TA)b | 1047053506529 | 75669..75701 | (TA)_n_ | TGAAGGAGATTCTCTGCGGT |
|  |  |  |  | CTCTCATCTTTTGTTGTGTCCG |
| 6559(CAA) | 1047053506559 | 94129-94191 | (CAA)_n_ | ATTCGGCCTGTTTGTATTCG |
|  |  |  |  | TTGGTTGTGTTTTACTGCTGC |
| 6559(GA) | 1047053506559 | 16332-16526 | (GA)_n_ | GCAAAAGCAACAAAAACAGC |
|  |  |  |  | GAGCACACGAAGGGGAATAA |
| 6559(TC) | 1047053506559 | 42683..42715 | (TC)_n_ | CGCTCTCAAAGGCACCTTAC |
|  |  |  |  | ATATGGACGCGTAGGAGTGC |
| 6559(TG) | 1047053506559 | 28781-28980 | (TG)_n_ | TTGCTTGCTTTCCACGTGTA |
|  |  |  |  | TATTCCCTTTGCCTTTGCTG |
| 6855(TC) | 1047053506855 | 76285-76410 | (TC)_n_ | GACATGTATGCTTGAAACCTCC |
|  |  |  |  | TCCATCTCCCTTCACACTCC |
| **6855(TTA)(GTT)** | 1047053506855 | 10052..10134 | (TTA)_n_(GTT)_n_ | GAGGTGATGACGATAAAATTGG |
|  |  |  |  | GTCTTTCCGCATATCCGAGA |
| **6855(TG)a** | 1047053506855 | 12867..12936 | (TG)_n_ | TTGCGTGGTTGTTTGTGC |
|  |  |  |  | GAGAAGAGGGGGAGGAAGAA |
| 6855(TA)(GA) | 1047053506855 | 5369-5576 | (TA)_n_(GA)_n_ | TGTGATCAACGCGCATAAAT |
|  |  |  |  | TTCCATTGCCTCGTTTTAGA |
| 6925(CT) | 1047053506925 | 88658-88832 | (CT)_n_ | CATCAAGGAAAAACGGAGGA |
|  |  |  |  | CGGTACCACCTCAAGGAAAG |
| 6925(TG)a | 1047053506925 | 119499..119520 | (TG)_n_ | TCGTTCTCTTTACGCTTGCA |
|  |  |  |  | TAGCAGCACCAAACAAAACG |
| 6925(TG)b | 1047053506925 | 48024..48053 | (TG)_n_ | GAAACGCACTCACCCACAC |
|  |  |  |  | GGTAGCAACGCCAAACTTTC |
| 7093(TC) | 1047053507093 | 69979..70016 | (TC)_n_ | CCAACATTCAACAAGGGAAA |
|  |  |  |  | GCATGAATATTGCCGGATCT |
| 7093(TA)c | 1047053507093 | 16233..16257 | (TA)_n_ | CGTGTGCACAGGAGAGAAAA |
|  |  |  |  | CGTTTGGAGGAGGATTGAGA |
| **7093(TAA)** | 1047053507093 | 82234-82445 | (TAA)_n_ | CCGCAGACATTTCTTCGACT |
|  |  |  |  | GCTTTTTGTCTTCTGCCGAC |
| 7093(TA)b | 1047053507093 | 51225-51373 | (TA)_n_ | GGAAACACATCACGCAAAGA |
|  |  |  |  | AGTGACAAAGGGGGACATTG |

| **Primer code** | **Contig ID^a^** | **Position^b^** | **Repeat type^c^** | **Forward/ Reverse primer (5'-3')** |
| --- | --- | --- | --- | --- |
|  |  |  |  |  |
| 10101(TAA)b | 1047053510101 | 987..1016 | (TAA)_n_ | CCGCGGTAGAAGAACCATAA |
|  |  |  |  | TGCGTATTCACGACGAGAAG |
| 10101(TA) | 1047053510101 | 46598..46638 | (TA)_n_ | AACCCGCGCAGATACATTAG |
|  |  |  |  | TTCATTTGCAGCAACACACA |
| 10101(TC) | 1047053510101 | 49478..49495 | (TC)_n_ | CGTACGACGTGGACACAAAC |
|  |  |  |  | ACAAGTGGGTGAGCCAAAAG |
| 10101(CA)a | 1047053510101 | 88534..88549 | (CA)_n_ | GTCGCCATCATGTACAAACG |
|  |  |  |  | CTGTTGGCGAATGGTCATAA |
| 10101(CA)c | 1047053510101 | 87584..87606 | (CA)_n_ | GTGTCGTTGCTCCCAAACTC |
|  |  |  |  | AAACTTGCCAAATGTGAGGG |
| 10101(CA)b | 1047053510101 | 15729..15742 | (CA)_n_ | ACCCAGAGGGGAGAAAAAGA |
|  |  |  |  | TTTACGGTTGGTTCGTGTGA |
| 10187(TG)b | 1047053510187 | 63326-63566 | (TG)_n_ | AAGAGAGGCACTCCCTGTGA |
|  |  |  |  | GAGGAAGAGGAAGTACAGTTGAGC |
| 10187(TG)a | 1047053510187 | 124985-125219 | (TG)_n_ | GCGCGTTATTAACACTCGCT |
|  |  |  |  | GCCCGGTATCATTGAAAAGA |
| 10187(TA) | 1047053510187 | 44002..44057 | (TA)_n_ | AGAAAAAGGTTTACAACGAGCG |
|  |  |  |  | CGATGGAGAACGTGAAACAA |
| 10187(GA) | 1047053510187 | 71097-71226 | (GA)_n_ | GTCACACCACTAGCGATGACA |
|  |  |  |  | ACTGCACAATACCCCCTTTG |
| 10187(TTA) | 1047053510187 | 32430-32629 | (TTA)_n_ | GAGAGAGATTCGGAAACTAATAGC |
|  |  |  |  | CATGTCCCTTCCTCCGTAAA |
| 10187(CA) | 1047053510187 | 83338-83417 | (CA)_n_ | CTACCTTCTCTTTCCTCCCTAACC |
|  |  |  |  | TTTGCTCTGGACTGCATGC |
| 10187(CA)(TA) | 1047053510187 | 39618-39874 | (CA)n(TA)_n_ | CATGTCATTAAGTGGCCACG |
|  |  |  |  | GCACATGTTGGTTGTTGGAA |
| 6789(TG) | 1047053506789 | 64001-64129 | (TG)_n_ | GAGCAGATCTTCCTTGTGCC |
|  |  |  |  | TGGTGAAATGCACGCATC |
| **6789(TC)** | 1047053506789 | 28533-28726 | (TC)_n_ | GCCCCCATGCATAATTTTTA |
|  |  |  |  | GTGGTGGGAGAACAACCAAC |

| **Primer code** | **Contig ID^a^** | **Position^b^** | **Repeat type^c^** | **Forward/ Reverse primer (5'-3')** |
| --- | --- | --- | --- | --- |
|  |  |  |  |  |
| 8741(TA) | 1047053508741 | 94443..94463 | (TA)_n_ | TGTAACGGTAGGTCTCAATTCG |
|  |  |  |  | TTGCACTTGTGTATCTCGCC |
| **8741(TC)** | 1047053508741 | 77851..77877 | (TC)_n_ | GCCATGTTTCCTTCACCAAC |
|  |  |  |  | AGGTGTTCCCCTCTTTGGAT |
| 8741(CT)(TA) | 1047053508741 | 72728-72864 | (CT)_n_(TA)_n_ | GCAGAGACGCACAGACACAT |
|  |  |  |  | AAAGTGCCATCCCACCCTC |
| **8741(TG)** | 1047053508741 | 47741..47761 | (TG)_n_ | AGAAGAGAGTCCGGAGTTTTC |
|  |  |  |  | TGTGCGCCAAGAGTTCTAAA |
| **8221(CA)** | 1047053508221 | 200539-200729 | (CA)_n_ | TCATTGTGTATGGGTGGTGG |
|  |  |  |  | CTGTTGGTCCTTTCAGTGCA |
| **10359(CA)(GA)** | 1047053510359 | 18473-18619 | (CA)_n_(GA)_n_ | AGTCCTACTGCCTCCTTGCA |
|  |  |  |  | ATTCATCGCCCGACATTTTA |
| **10359(TTA)** | 1047053510359 | 15248-15407 | (TTA)_n_ | GCAATTGCTGCTTTCGGTAT |
|  |  |  |  | TCACGCTGCCGTACAAGTAG |
| 11283(A)(CA) | 1047053511283 | 58996-59147 | (A)_n_(CA)_n_ | GCGGTACACCAACATGTACG |
|  |  |  |  | GTGTGTTTGTGTGTGAGAGGC |
| **11283(TA)a** | 1047053511283 | 17391..17437 | (TA)_n_ | CCAGTATTCCTCCCCCCTAA |
|  |  |  |  | CGTCTTGTTGATTTCCCCTT |
| 11283(TCG) | 1047053511283 | 88680..88708 | (TCG)_n_ | ACCACCAGGAGGACATGAAG |
|  |  |  |  | TGTACACGGAACAGCGAAG |
| 11283(TA)b | 1047053511283 | 12639-12819 | (TA)_n_ | AACATCCTCCACCTCACAGG |
|  |  |  |  | TTTGAATGCGAGGTGGTACA |
| **11863(CA)** | 1047053511863 | 7458..7500 | (CA)_n_ | AGTTGACATCCCCAAGCAAG |
|  |  |  |  | CCCTGATGCTGCAGACTCTT |
| mclf10** | Unknown | Unknown | (CA)_n_A(CA)_n_ | GCGTAGCGATTCATTTCC |
|  |  |  |  | ATCCGCTACCACTATCCAC |
| **Set0** | Unknown | Unknown | Unknown | CCTCTGCGCACACATTCATT |
|  |  |  |  | CCGTTCTTCATCACCATCCT |
| scle10** | Unknown | Unknown | (CT)_n_(TG)_n_ | GATCCCGCAATAGGAAAC |
|  |  |  |  | GTGCATGTTCCATGGCTT |
| TcUn2 | Unknown | Unknown | Unknown | AACAAAATCTAGCGTCTACCATCC |
|  |  |  |  | GGTGTTGGCGTGTATGATTG |
| TcUn4 | Unknown | Unknown | Unknown | CAGAAACGTCAAGTACGGAGC |
|  |  |  |  | ATCCCCTTTAATAGCGAGCG |
| TcUn1 | Unknown | Unknown | Unknown | ATGCTCCGCAACATATTACTCA |
|  |  |  |  | GTCGAGCTTCTGTTGTTCCC |

^a^ Refers to a sequence fragment identified by the *T. cruzi* genome project (www.**tigr**.org).

^b^ Refers to the position of the of the primer binding site in the flanking region 5’-3’ along the sense strand. Unidentified positions are marked ‘unknown’.

^c^ Refers to the repeat type (e.g. di-nucleotide, tri-nucleotide). Unidentified repeat types are marked ‘unknown’.

** mclf10 and scle10 published in Oliveira et al., (1998)

Primer codes in bold correspond to those which are unique to this study.
